# Age-related changes in T lymphocytes of patients with head and neck squamous cell carcinoma

**DOI:** 10.1186/s12979-020-0174-7

**Published:** 2020-02-12

**Authors:** S. S. Jeske, P. J. Schuler, J. Doescher, M. N. Theodoraki, S. Laban, C. Brunner, T. K. Hoffmann, M. C. Wigand

**Affiliations:** Department of Oto-Rhino-Laryngology, Head and Neck Surgery, Ulm University Medical Center, Frauensteige 12, 89075 Ulm, Germany

**Keywords:** Head and neck cancer, Aging, T cells, Immunosenescence, Immune escape

## Abstract

**Introduction:**

The number of aging cancer patients has increased continuously and will do so further in the future. The immune system of elderly people experiences critical changes over the time. Therefore, tumor-induced changes in the immune system are believed to differ in young and elderly cancer patients as well.

**Methods:**

The effect of aging on the immune system was measured in peripheral blood lymphocytes (PBL) of healthy volunteers (*n* = 48, 21–84 yrs.) divided into three different age groups. Seventy years was set as a cut-off for defining subjects as elderly. Results were compared to two groups of adult cancer patients, which donated PBL and tumor infiltrating lymphocytes (TIL): young cancer patients (40–69 yrs.; blood: *n* = 13; TIL: *n* = 17) and elderly cancer patients (70–90 yrs.; blood: *n* = 20; TIL: *n* = 15) with head and neck squamous cell carcinoma (HNSCC). Frequencies and phenotypes of CD4^+^ and CD8^+^ T cells as well as regulatory T cells (T_reg_) were assessed by flow cytometry.

**Results:**

We observed lower frequencies of CD8^+^ cytotoxic T cells during aging in both groups. Frequencies of tumor infiltrating regulatory T cells were significantly higher than in the peripheral blood but showed a significant decline in older tumor patients. With increasing age, expression of immunosuppressive CD73 and CCR7 was lower and expression of PD1 elevated on peripheral T cells in healthy volunteers and tumor patients.

**Conclusion:**

Immunosenescence takes place in healthy donors and cancer patients. Our results suggest that in elderly tumor patients, the immune system is impaired and the tumor-induced immune escape is less pronounced. The increased expression of PD1 implies the potential for effective immunotherapies in elderly, as treatment with checkpoint inhibitors could be more beneficial for elderly HNSCC patients.

## Introduction

Population ageing has become one of the most significant sociological and medical issues of the twenty-first century. According to data from ‘World Population Prospects’ [1], the population aged 60 or above is growing faster than all younger age groups, globally. While this population group counted 962 million people in 2017, it is estimated to rise up to 2.1 billion by 2050 and up to 3.1 billion by 2100. Besides socioeconomic issues, a growing and ageing society constitutes an immense public health burden. As it is the case for almost every malignancy, the number of older patients suffering from head and neck squamous cell carcinoma (HNSCC) has increased in the past decade and is projected to rise further in the future [2]. Despite this development, there exist only few studies concentrating on this patient subgroup. In fact, it has been under-represented in many influential studies, which have been of significant impact on standard-of-care guidelines [3]. However, carcinogenesis in older patients requires a different angle of view as the immune system undergoes a wide range of changes with increasing age. Both the innate and the adaptive immune system are affected by transformations of their constituents and functions, generally referred to as immunosenescence [4, 5]. The numerous alterations are contributing to not only increased susceptibility to infectious diseases and decreased response to vaccination but also to carcinogenesis in older people [6]. Against the background of diverse alterations in the immune system of elderly persons, our focus of interest lies in the adaptive immune system and in T lymphocytes in particular.

With increasing age, the thymus, a primary lymphoid organ, where T cells mature, shrinks constantly and gets replaced by fat tissue. Consequently, the number of naïve T lymphocytes exiting the thymus decreases steadily. This process can be observed especially for CD8^+^ T cells [7]. As a result of the reduced output of naïve T lymphocytes, a relative increase of functionally exhausted memory and effector cells occurs with increasing age [8, 9].

After maturing in the thymus, naïve T lymphocytes are transported to lymph nodes, where antigen presentation as well as differentiation and proliferation of T lymphocytes takes place. C-C chemokine receptor type 7 (CCR7) is particularly involved in the ‘homing’ of T lymphocytes and dendritic cells to lymph nodes and has been reported to protect effector T cells from apoptosis. In HNSCC, the expression of this molecule has been found to be increased on tumor cells, which seems to be associated with the development of lymph node metastases and disease progression [10, 11]. Its expression on CD8^+^ T cells, on the other hand, appears to decrease in HNSCC patients with lower CD8^+^CCR7^+^ frequencies predicting disease recurrence [12]. Kim et al., postulated that CCR7 protects effector T cells from apoptosis and that its reduced expression on CD8^+^ T cells in HNSCC contributes to apoptosis and a rapid turnover of these cells [13]. Due to its relevance for disease progression, age-related changes in the expression of CCR7 could thus contribute to carcinogenesis in elderly patients.

The presence of tumor infiltrating lymphocytes (TIL) in the tumor microenvironment is known to have a relevant impact on prognosis in different types of cancer. However, it is necessary to distinguish carefully between the different subtypes of lymphocytes as they may have different and even conflicting functions [14]. Tumor-infiltrating CD8^+^ T cells, for example, act cytotoxic and have been shown to be associated with a better prognosis in different malignancies including HPV-positive and -negative HNSCC [14–16]. Tumor-infiltrating regulatory T cells, on the other hand, support the immunosuppressive milieu and inhibit immune response towards tumor cells. They have been observed to accumulate in aged mice and to be correlated with immune deficiency [17]. We were hence interested if we could detect age-related changes in tumor-infiltrating T cells in our HNSCC patient group as well.

HNSCC, in general, is regarded as an immunogenic tumor with favorable response to immunotherapeutic approaches, especially to treatment with checkpoint inhibitors [18]. One of their major targets represents the checkpoint molecule programmed cell death 1 (PD1) which promotes the immunosuppressive milieu of solid tumors. In HNSCC, PD1 has been shown to be increased both on circulating and intratumoral T cells with a significantly higher rate of PD1 on tumor-infiltrating T cells [19]. The authors proposed that this may reflect the immunosuppressive environment in HNSCC and point towards higher proportions of regulatory or exhausted T-cell phenotypes. Despite the growing number of geriatric tumor patients in clinical practice, this patient subgroup has been addressed in few clinical studies on PD1 inhibitors. To date, it seems as if age-related treatment response depends on the tumor entity. In a meta-analysis on more than 11,000 patients suffering from different tumors such as non-small cell lung cancer, melanoma, gastric, renal and urothelial cancer, Wu et al. stated that patients aged 65 and more years seem to benefit more from immunotherapy than younger patients [20]. Interestingly, in a recent study investigating the response of melanoma patients to PD1 inhibition, patients over the age of 60 responded significantly better to treatment with the anti-PD1 antibody pembrolizumab than younger patients [21]. With regards to HNSCC, age-related response rates to immunotherapy have not yet been finally clarified as only few older patients have been included into clinical trials. Against this background, we were particularly interested to examine, if differences in PD1 expression can be observed in older HNSCC patients.

Finally, it was our aim to investigate, if age has an influence on the expression of the adenosine (ADO) producing ectonucleotidases CD39 and CD73 due to our previous focus on these markers [22–24]. Adenosine has an immunosuppressive effect in the tumor micro-environment and age-related changes of CD39 and CD73 could therefore affect anti-tumor immune response in the elderly.

To sum up, the following study aimed to investigate differences in T-cell subgroups and their expression profile with increasing age both in healthy subjects and young and elderly patients who suffered from HNSCC.

## Material and methods

### Blood samples

Peripheral blood lymphocytes (PBL) were obtained from treatment-naïve tumor patients (TB; *n* = 33, 47–90 yrs) and healthy volunteers (*n* = 48, 21–84 yrs). All participants signed an informed consent form approved by the local ethics committee (#255/14). Blood (50 ml) was collected in S-monovettes prefilled with trisodium citrate (Sarstedt) and centrifuged on Biocoll Separating Solution (Merck) or Pancoll (density: 1119 g/ml and 1077 g/ml; PAN Biotech). PBL were recovered, washed twice, and stored for further experiments in liquid nitrogen. Subjects were divided in five groups, predefined by their diagnosis and age (group 1: healthy subjects, 21–39 years; group 2: healthy subjects, 40–69 years; group 3: healthy subjects, 70–90 years; group 4: tumor patients, age 40–69 years; group 5: tumor patients age 70–90 years). Seventy years was used as cut-off for defining patients as elderly in accordance to numerous other current studies [25–27]. Clinicopathological and demographic parameters are listed in Table 1.

**Table 1:** Clinicopathological parameters of the patients’ blood

	Group 1	Group 2	Group 3	Group 4 blood	Group 5 blood	Group 4 TIL	Group 5 TIL
Mean age (range)	26.1 (21–35)	51.4 (40–67)	75.6 (70–84)	58.2 (47–65)	79.1 (70–90)	57.9 (46–68)	79.4 (70–90)
Gender							
Male	10	12	10	9	16	13	13
Female	5	5	6	4	4	4	2
T classification							
T1				0	6	0	5
T2				3	1	4	0
T3				5	6	6	5
T4				5	6	5	5
Tx				0	1	2	0
N classification							
N0				1	11	1	7
N1				3	4	5	4
N2				8	3	9	3
N3				1	2	2	1
M classification							
M0				13	16	16	12
M1				0	3	1	3
Mx				0	1	0	0
Death				5	7	7	6
HPV-status							
Positive				2	0	2	0
Localization							
Pharynx				9	5	10	6
Larynx				2	6	2	4
Mouth				1	4	2	1
Nose				1	0	1	0
CUP				0	1	2	0
Other				0	4	0	4

### Tumor infiltrating lymphocytes

Tumor tissue (TT) of the same treatment-naïve patients was collected directly after surgery in NaCl, whenever possible (*n* = 32, 46–90 yrs). Minced tissue pieces were collected in RPMI medium (Gibco) containing 200 IU/ml Collagenase I (Worthington) for at least 2 h at 37 °C. After digestion the tissue was mashed with a 100 μm EASY strainer (Gibco). The lymphocyte fraction was isolated via Pancoll centrifugation. The purity of the cells was measured by anti-CD45 staining. Clinicopathological and demographic parameters are listed in Table 1 as well.

### Antibodies and reagents

The following anti-human monoclonal antibodies (mAbs) were used for flow cytometry: CD4 Alexa Fluor® 700, CD8 APC, CD39 PE-Cy7, CD73 FITC, and CD73 eFluor450, CCR7 (CD197) PE-Cy7, PD1 (CD279) PE (eBioscience); CCR7 PE-CF594, CD45 AmCyan and CD45 FITC (Becton Dickinson), CD3 APC-H7, CD25 FITC (BioLegend) and CD25 PE (MACS Miltenyi). All mAbs were titrated using normal PBL to establish optimal dilution. T_reg_ were defined by their ability to produce extracellular adenosine as previously described by Deaglio and Borselino [28, 29]. We have previously demonstrated that these CD4^+^ CD39^+^ CD25^+^ T_reg_ are FOXP3^+^ CD127^neg^ [24].

### Surface staining

Cells were incubated with mAbs at RT for 30 min in the dark, then washed and collected in PBS for flow cytometry analysis. All flow cytometry measurements were performed using a Gallios 10-color-flow-cytometer equipped with Kaluza flow cytometry software (both Beckman Coulter). The acquisition and analysis gates were restricted to the lymphocyte gate based on characteristic properties of the cells in forward and side scatter. At least 10^5^ cells were acquired for the analysis.

### T-cell culture

PBL were cultured in 96-well plates (2 × 10^5^ cells/well) containing RPMI + 10% FBS superior (Merck, USA), 100 U/ml penicillin and 100 μg/ml streptomycin (Pan Biotech, Germany). Cells were stimulated with anti-CD3 (1 μg/ml, BD) and three concentrations of anti-CD28 (1 μg/ml, 5 μg/ml or 10 μg/ml; BD) and cultured for at least 7 days (37 °C, 5%CO_2_). FACS analysis of CD73 on the T-cell subpopulations was performed as described above.

### Statistical analysis

All data are presented as means of at least three experiments. Data were tested for Gaussian distribution using Shapiro-Wilk test. One-Way ANOVA or Kruskal-Wallis test were used to test for group differences as appropriate. Mann-Whitney test was applied for non-parametric parameters. For correlation analyses, Pearson and Spearman test were used for parametric and non-parametric values, respectively. The significance value for *p*-values was set at ≤0.05. All statistical analyses were performed using GraphPad Prism Version 6.01.

## Results

### Age-related changes in lymphocyte subsets

**Fig. 1:**
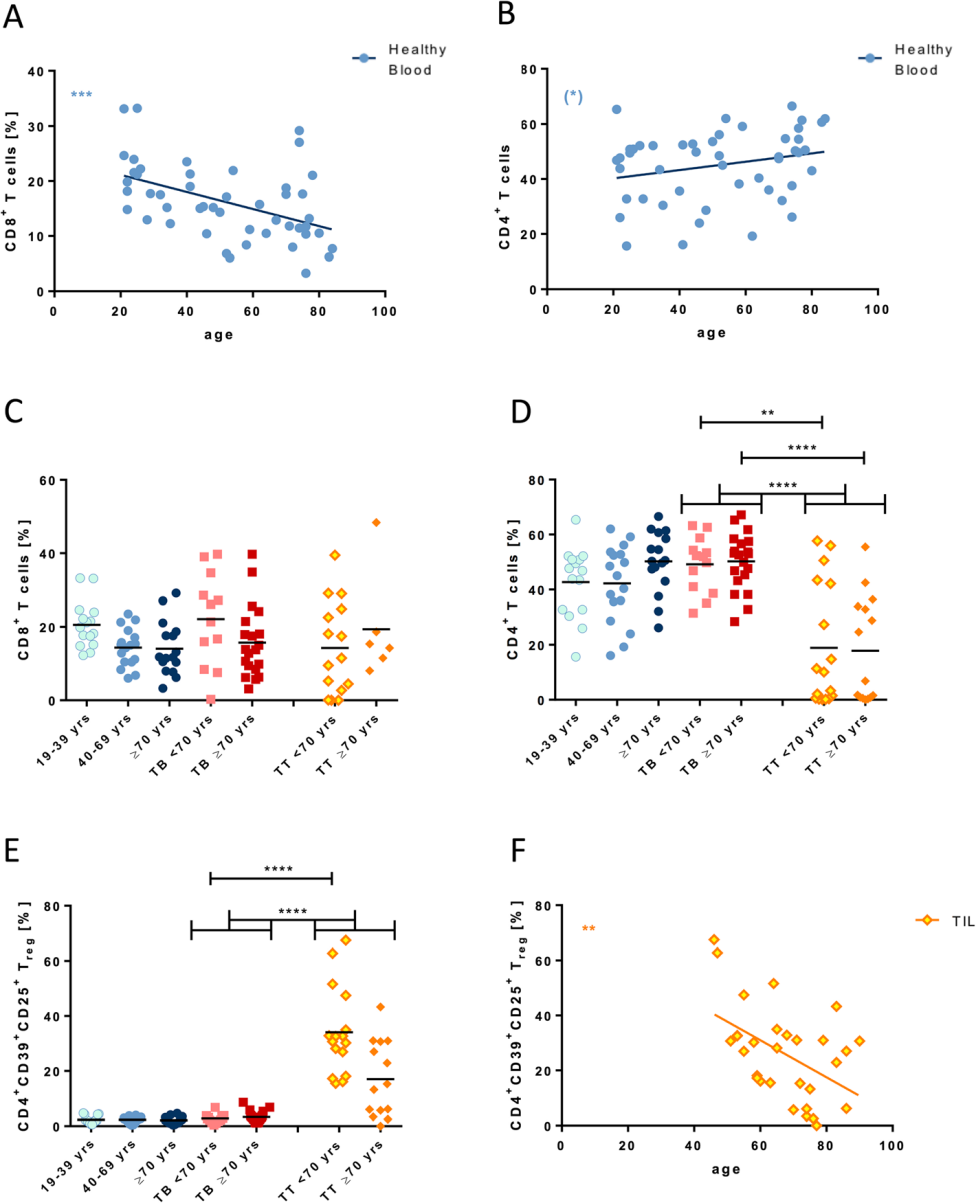
Lymphocyte populations of different ages from healthy donors and HNSCC patients. Correlations between frequencies of CD8^+^ (**a**) and CD4^+^ T cells (**b**) and age in healthy donors. Frequencies of CD8^+^ (**c**), CD4^+^ T cells (**d**) and T_reg_ (**e**) in healthy donors and tumor patients of different age groups (healthy < 40: light blue; healthy > 40 and ≤ 70: blue; healthy ≥70: dark blue; tumor blood (TB) < 70: light red; tumor blood ≥70: red; TIL (TT) < 70: yellow; TIL ≥ 70: orange). Cell populations were plotted in a regression line (**a-b**, **f**) (healthy: blue circles; TIL: yellow rhombs). All data are plotted showing the mean. *P* < 0.01 (**); *p* < 0.001 (***); *p* < 0.0001(****)

In healthy donors, a negative correlation between age and the frequency of CD8^+^ cytotoxic T (T_c_) cells was observed (*p* < 0.001, Fig. 1a) while the frequency of CD4^+^ T helper (T_h_) cells remained relatively stable (*p* < 0.1, Fig. 1b). Probably due to the limited number of samples, this effect could not be reproduced, when the predefined age groups were compared (Fig. 1c and d). We saw a significant positive correlation between age and the CD4/CD8 ratio in healthy subjects (*p* < 0.001, Additional file 1: Figure S1A). The frequency of regulatory T cells (T_reg_) in the blood remained stable for all age groups (Fig. 1e). In healthy donors, we observed a negative correlation between peripheral CD4^+^ T_h_ cells and T_reg_ (*p* < 0.01, *R*^*2*^ = 0.2; Additional file 1: Figure S1B), indicating that the unchanged proportion of T_reg_ is not affected by a lower CD4^+^ T_h_ cell frequency.

In tumor patients’ blood (TB), the frequency of CD8^+^ T cells showed a slight decrease in elderly patients similar to our observations in healthy volunteers (*p* = 0.08, Fig. 1c). The frequency of CD4^+^ T cells and T_reg_ remained stable for young and elderly cancer patients (Fig. 1d and e). In tumor tissue (TT) samples, the frequency of CD8^+^ TIL was comparable to PBL of cancer patients with no significant age-related changes (Fig. 1c). However, the frequency of infiltrating CD4^+^ T cells was significantly lower (*p* < 0.0001) than in PBL, and remained stable with increasing age (Fig. 1d). Interestingly, T_reg_ showed significantly higher frequencies in tumor tissue as compared to blood samples (Fig. 1e). However, the frequency of tumor-infiltrating T_reg_ decreased in elderly cancer patients as compared to young tumor patients and showed an inverse correlation to age (*p* < 0.01, *R*^*2*^ = 0.2; Fig. 1e and f). Despite this decline, the frequency of tumor-infiltrating T_reg_ in elderly tumor patients was still significantly higher than in the peripheral blood. As there were many possible confounders for altered frequencies of TIL subsets, we tested for differences in tumor site, T stage, HPV status and gender by setting these parameters as confounders in separate ANOVA tests. The tests showed that the observed results were not confounded by any of the stated factors (data not shown). Additionally, the total numbers of lymphocytes were analyzed in all blood samples. We observed an age-dependent decrease of CD8^+^ T_c_ cells in the peripheral blood of healthy volunteers (*p* < 0.001, *R*^*2*^ = 0.1), which was not seen in cancer patients (Additional file 1: Figure S1C). The decrease of CD8^+^ T_c_ cells was also seen for the age group ≥70 years in age group comparisons (*p* < 0.05, Additional file 1: Figure S1D). No such age-dependent correlations were detected for CD4^+^ T_h_ cells or T_reg_ (Additional file 1: Figure S1E and F). Incidentally, no group differences were detected, when cell counts of total lymphocytes were analyzed.

### Expression of the ectonucleotidases CD39 and CD73

**Fig. 2:**
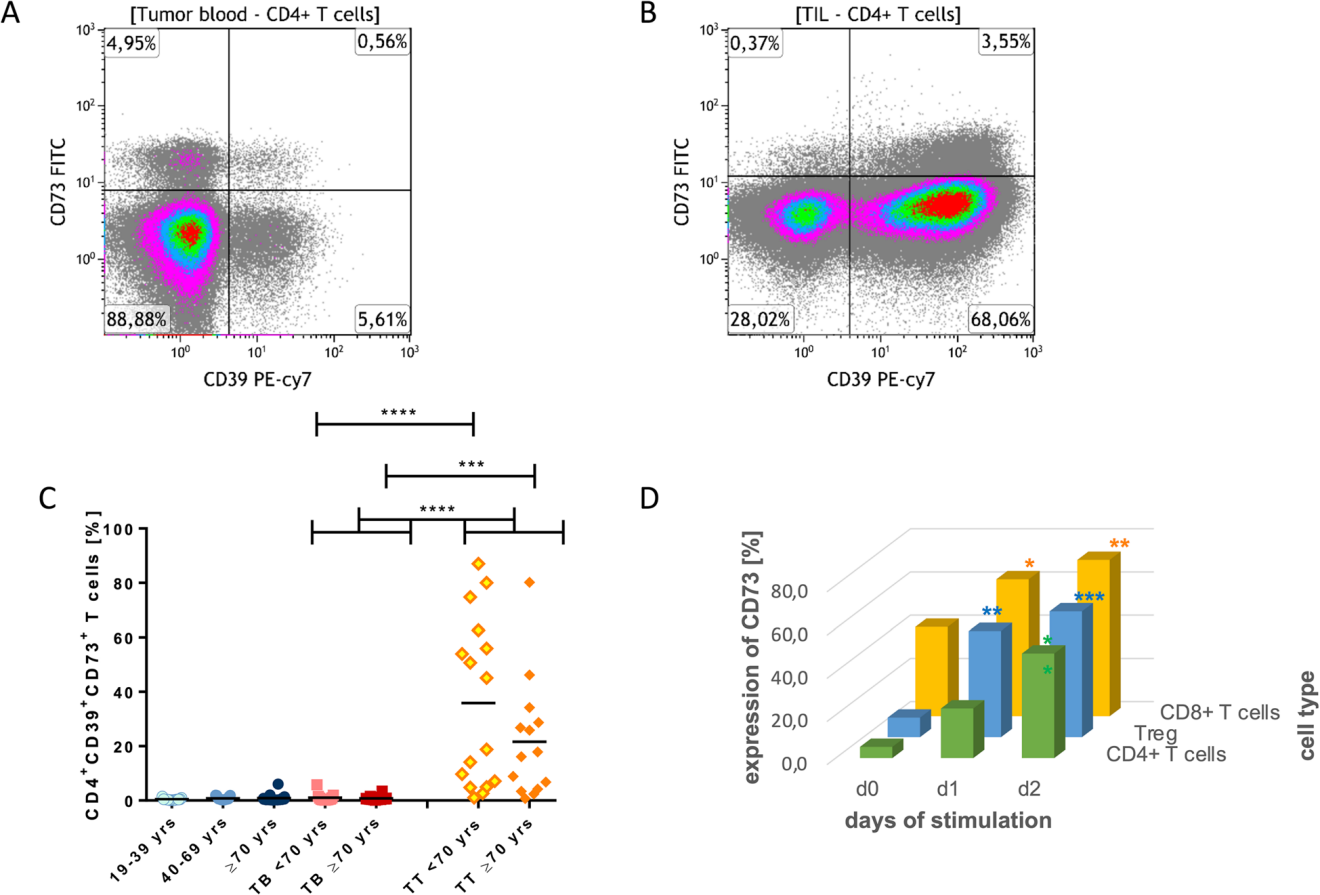
Expression levels of the ectonucleotidases CD39 and CD73 on CD4^+^ T cells in healthy donors and HNSCC patients in comparison as well as CD73 expression in response to T-cell stimulation. PBL were examined for surface expression of the ectonucleotidases CD39 and CD73 by FACS (**a-b**). The CD39 and CD73 characterization of CD4^+^ T cells of the healthy and tumor blood in representative density plots. The co-expression of CD39 and CD73 on CD4^+^ blood T cells were plotted for healthy donors and tumor patients and compared to CD4^+^ TIL (**c**). PBL were incubated with anti-CD3 and anti-CD28 for at least 2 days. The frequency of CD73 on CD8^+^, T_reg_ and CD4^+^ T cells was measured by FACS before and after each day of stimulation (*n* = 4) (**d**). Data were analyzed with a Two-Way ANOVA and shown as mean; *p* < 0.05 (*); *p* < 0.01 (**); *p* < 0.001 (***)

To further characterize the T cell subpopulations, we analyzed the expression of the adenosine (ADO) producing ectonucleotidases CD39 and CD73, which can define a variety of regulatory cell populations [22, 23, 30]. CD4^+^ T_h_ cells of the blood can be divided into three subpopulations based on their expression of CD39 and CD73: (I) CD39^+^ T_reg_, (II) activated CD73^+^ T_h_ cells, and (III) double negative T_h_ cells (Fig. 2a). As described before, the co-expression of CD39 and CD73 is extremely rare in peripheral CD4^+^ T_h_ cells [24]. In contrast, the frequency of CD4^+^CD39^+^ T_reg_ (*p* < 0.05; Fig. 2B) and of double-positive CD4^+^CD39^+^CD73^+^ T cells (*p* < 0.0001; Fig. 2C) was increased in the tumor tissue as compared to peripheral blood.

**Fig. 3:**
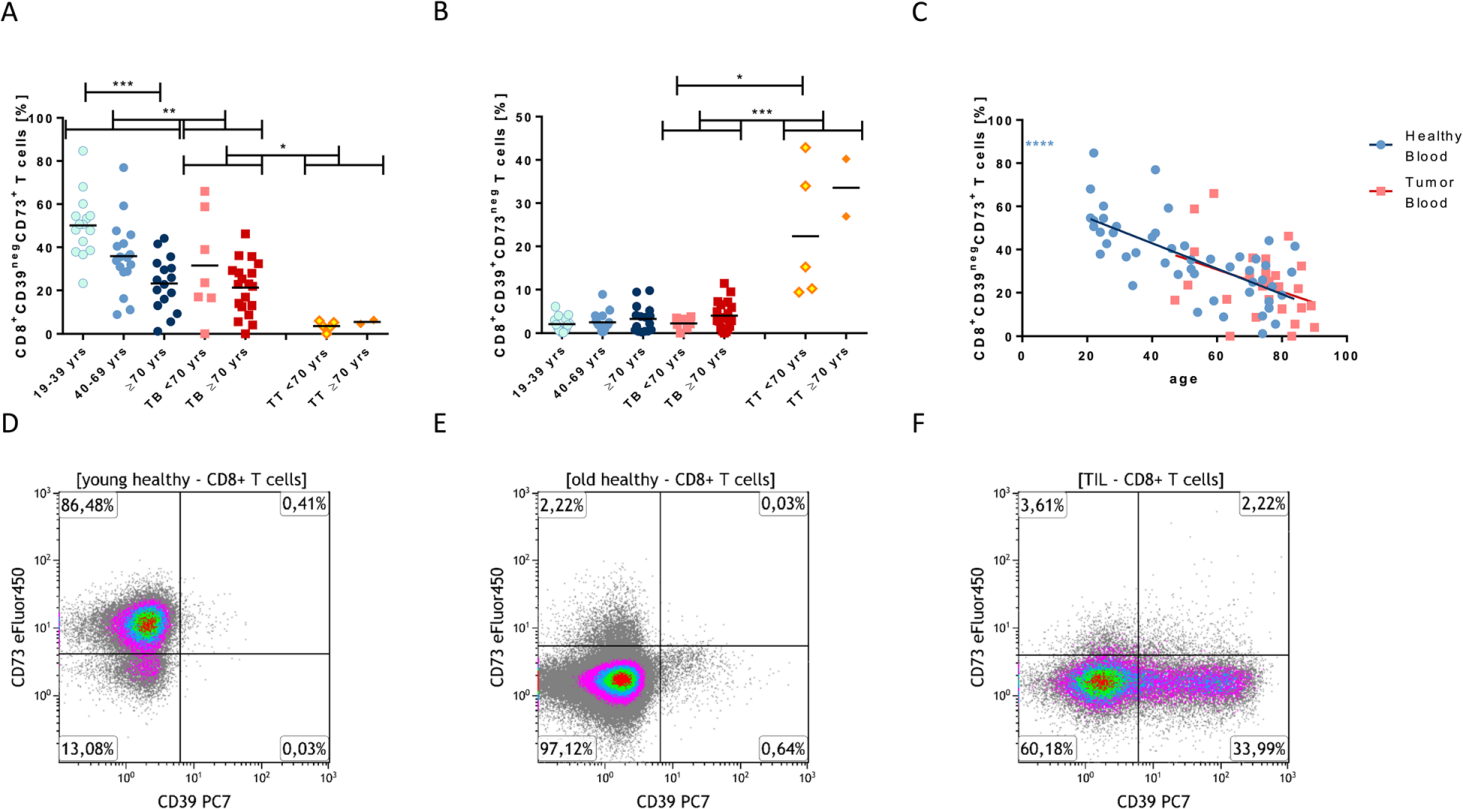
Expression levels of CD39 and CD73 on CD8^+^ T cells in healthy donors and HNSCC patients in comparison. The expression levels of CD8^+^CD39^neg^CD73^+^ (**a**) and CD8^+^CD39^+^CD73^neg^ (**b**) in the different groups were plotted in. The trend of CD8^+^CD39^neg^CD73^+^ T cells of tumor and healthy blood cells over increasing ages is plotted in (**c**) (tumor blood: red squares). Representative density plots for CD8^+^ healthy donors of different ages (**d/e**) and TIL (**f**). All data are plotted showing the mean or the linear regression. *P* < 0.05 (*); *p* < 0.01 (**); *p* < 0.001 (***); *p* < 0.0001(****)

Interestingly, the expression of CD73 was significantly lower (*p* < 0.0001) on peripheral CD8^+^ T cells of older healthy volunteers, while CD39 was nearly not expressed on peripheral CD8^+^ T cells (Fig. 3a-e). The age-dependent decline of CD73 seems to persist in cancer patients, although this observation did narrowly miss statistical significance (*p* = 0.05). We assumed that the enzyme CD73 is not only responsible for the production of ADO, but also a marker for cell activity. Accordingly, our data show that the expression of CD73 increases after stimulation of T-cell subpopulations in a time-dependent manner (*p* < 0.01, Fig. 2d). We also tested different concentrations of CD28 from 1 to 10 μg/ml, but saw no differences (data not shown). After prolonged stimulation, the initial expression of CD73 diminished on days 3 to 7, possibly due to cell fatigue (data not shown). We were surprised to see that the expression of the ectonucleotidases CD39 and CD73 was reversed on tumor-infiltrating CD8^+^ T_c_ cells, which are mostly CD39^+^CD73^neg^ in comparison to peripheral CD8^+^ T_c_ cells, which are mainly CD39^neg^CD73^+^ (Fig. 3a and b).

### Age-related increase of PD1

**Fig. 4:**
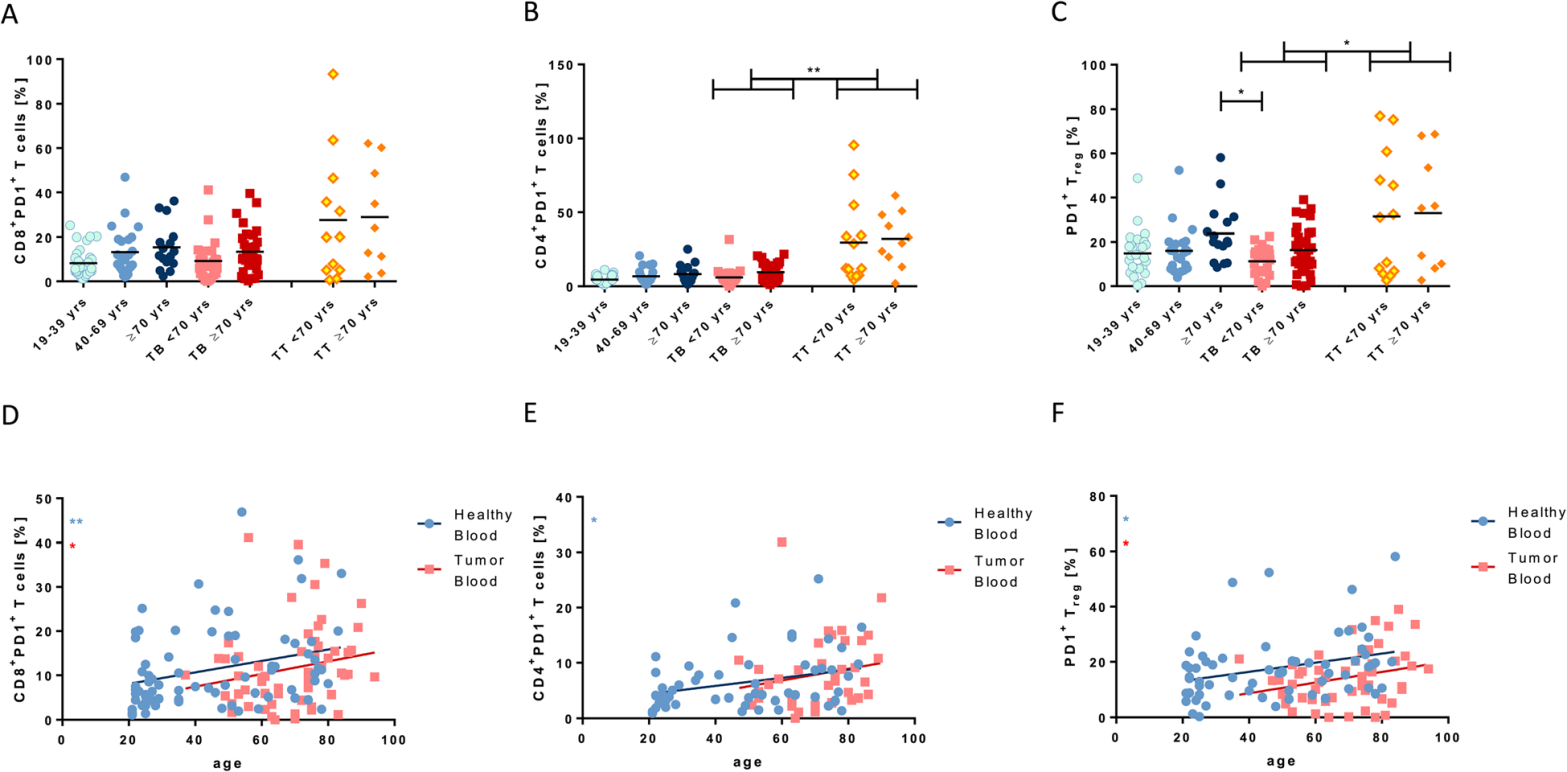
PD1 expression on T cell subtypes. The expression levels of PD1 on CD8^+^ T cells (**a**), CD4^+^ T cells (**b**) and T_reg_ (**c**) in the different groups were plotted. The trend of the expression of PD1 on CD8^+^ T cells (**d**) and CD4^+^ T cells (**e**), as well as for T_reg_ (**f**) for both healthy and tumor blood volunteers was depicted. P < 0.05 (*); *p* < 0.01 (**)

All T cell subpopulations showed low levels of PD1 expression on their surface (~ 10%), while all TIL populations expressed significantly higher PD1 levels than tumor blood cells (Fig. 4a-c). In healthy volunteers, PD1 expression showed a positive correlation with age on all T cell subpopulations (CD8^+^ T_c_ cells: *p* < 0.01, *R*^*2*^ = 0.08; CD4^+^ T_h_ cells: *p* < 0.05, *R*^*2*^ = 0.09; T_reg_: *p* < 0.05, *R*^*2*^ = 0.09; Fig. 4d-f). The same correlation was observed in the blood of tumor patients (CD8^+^ T_c_ cells: *p* < 0.05, *R*^*2*^ = 0.03; CD4^+^ T_h_ cells: *p* < 0.1, *R*^*2*^ = 0.04; T_reg_: *p* < 0.05, *R*^*2*^ = 0.07; Fig. 4d-f).

### Age-related decrease of CCR7

**Fig. 5:**
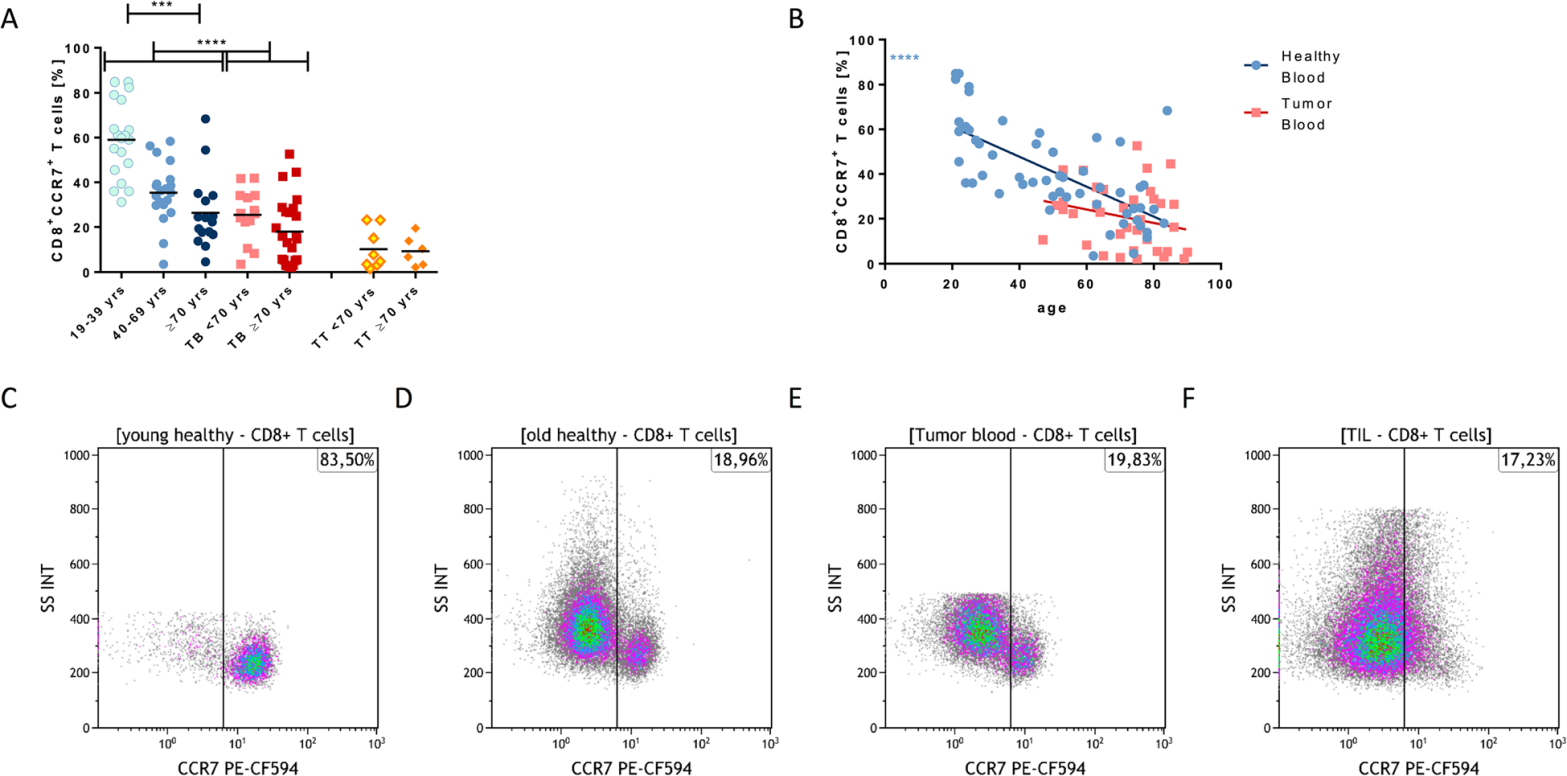
CCR7 expression on CD8^+^ T cells. The expression of CCR7 on CD8^+^ T cells (**a**) for the different aging groups and the development of CCR7 on CD8^+^ T cells (**b**) are plotted in comparison of healthy and tumor blood T cells. **c-f** The CCR7 characterization of CD8^+^ T cells of healthy young (**c**) and old donors (**d**), the tumor blood (**e**) and the phenotype of tumor infiltrating CD8^+^ T cells (**f**) in exemplary density plots. All data are plotted showing the mean or the linear regression. *P* < 0.001 (***); *p* < 0.0001 (****)

The expression of CCR7 on CD8^+^ T_c_ cells dropped significantly with age in healthy volunteers (*p* < 0.0001; Fig. 5a and b). Interestingly, expression of CCR7 was even lower on CD8^+^ T_c_ cells of cancer patients (Fig. 5a). However, this observation may be partly biased by the increased mean age in the cancer patient cohort. A further decrease of CCR7 expression was observed in tumor-infiltrating CD8^+^ T_c_ cells when compared to peripheral blood of tumor patients (*p* = 0.06; Fig. 5a). Exemplary density plots for CCR7 expression on CD8^+^ T_c_ cells are shown in Fig. 5c-f. CCR7 expression on CD4^+^ T cells of healthy subjects and tumor patients remained stable over age, but its expression was significantly lower in tumor patients (*p* < 0.01) as compared to healthy volunteers and in TIL compared to PBL of tumor patients (*p* < 0.05) (Additional file 2: Figure S2A and B). In T_reg_, no differences were seen for CCR7 expression with data not being available for all tumor samples (Additional file 2: Figure S2C and D). The co-expression of PD1 and CCR7 on all observed T cell populations was higher on TIL when compared to peripheral blood T cells (data not shown). The low levels of CCR7 on CD4^+^ and CD8^+^ T cells in the tumor tissue compared to the blood of tumor patients are shown in representative density plots (Additional file 2: Figure S2E).

## Discussion

Our data reveal numerous age-dependent alterations of T-cell populations both in healthy controls and in cancer patients. Especially on CD8^+^ T_c_ cells, we found a number of changes due to aging, which demonstrate immunosenescence in elderly healthy volunteers, including (I) decreased frequency and absolute number in the peripheral blood, (II) decreased cell activity measured by CD73 expression, (III) increased expression of the checkpoint PD1, and (IV) decreased CCR7 expression. We hypothesize that the mechanisms of tumor-induced immune suppression may be less pronounced in elderly patients with the remaining ones being targetable (PD1). The decreased tumor-induced immune suppression is indicated by a decreased frequency of tumor-infiltrating T_reg_ and a decreased production of extracellular ADO by CD73^+^ immune cells (Additional file 3: Figure S3).

### Frequency and number of T_c_ cells

Both healthy subjects and tumor patients showed an age-related decrease in CD8^+^ T_c_ cells regarding frequency and total numbers in our cohort (Fig. 1a and c, Additional file 1: Figure S1C and D). As the size of the thymus – the place where T lymphocytes mature – decreases significantly with age, the number of naïve T lymphocytes exiting the thymus is reduced in older subjects [7]. Accordingly, the frequency of naïve T cells decreases, while the frequency of memory and effector cells increases with age. As described by Saavedra et al., the number and proportion of naïve CD8^+^ lymphocytes may be reduced due to a decreased output of naïve lymphocytes with increasing age while terminally differentiated CD8^+^ T cells accumulate [31]. The frequency of tumor-infiltrating CD8^+^ T_c_ cells, on the other hand, remained stable in our cohort of elderly cancer patients. In general, higher counts of intratumoral CD8^+^ T_c_ cells have been related to a better prognosis in different types of cancer including HNSCC [14–16]. In addition, we observed a positive correlation between the CD4^+^/CD8^+^ T-cell ratio and age in our healthy subjects (Additional file 1: Figure S1A), which is supported by the data of others [32]. This age-related increase of the CD4^+^/CD8^+^ T-cell ratio was not seen in our group of cancer patients. Remarkably, in lung cancer patients, an increased CD4^+^/CD8^+^ T-cell ratio has been associated with a better response to an experimental EGF cancer vaccine [33]. In nasopharyngeal carcinoma, patients with higher CD4^+^/CD8^+^ T-cell ratios showed a better survival than patients with lower ratios [34]. This positive effect of an elevated CD4^+^/CD8^+^ T-cell ratio seems to be compromised in those patients suffering from cancer in our cohort which might point towards a more disadvantageous composition of the immune system in the latter. It seems as if disturbances in the physiological age-related changes in the T cell system are at least partially associated with carcinogenesis.

### Expression of ectonucleotidases CD39 and CD73

Our data show that the enzyme CD73 not only is responsible for the production of ADO [23], but also acts as an activity marker, as it increases after stimulation on all T-cell subpopulations, in vitro (Fig. 2d). Interestingly, the expression of CD73 on CD8^+^ T_c_ cells showed a negative correlation with age in healthy individuals and in our small cohort of tumor patients (Fig. 3c). This may be indicative for a decreased activity of CD8^+^ T_c_ cells in elderly subjects. As the immune cells become less active with increasing age, there may be a decreased need for the production of immunosuppressive ADO in the blood and tissue of elderly individuals, which is partly regulated by the expression CD73. Although, this hypothesis is intriguing, we cannot support it by experimental data so far.

### Expression of checkpoint PD1

The checkpoint PD1 is an immune checkpoint molecule, which is expressed on a variety of immune cells. Activation of PD1 induces a decreased activity of these cells [35]. High expression of PD1 may, therefore, be linked to a decreased activation status of the immune system.

Interestingly, PD1 expression increased with age on all T cell subpopulations both in healthy volunteers and in tumor patients indicating a shift towards increased immunosuppression in older subjects (Fig. 4). It is therefore reasonable, that elderly cancer patients show a better response to clinical PD1-inhibition than younger patients, as shown by Kugel et al. in a cohort of melanoma patients [21]. Regarding HNSCC, current studies on the treatment response of older tumor patients after immunotherapy are still rare. The Keynote-012 trial on the efficacy and safety of pembrolizumab in recurrent or metastatic HNSCC revealed an overall response rate of 18% with the average age of patients being 63 years [36]. No data on possible correlations between age and treatment response were found in this study. The Checkmate-141 trial compared the efficacy of nivolumab with standard, single-agent systemic therapy on HNSCC patients with progressive recurrent disease within 6 months after platinum-based chemotherapy [37]. Initially, patients between 65 and 75 years showed no differences between the two treatment arms, while the overall survival of younger patients was significantly longer with nivolumab. A second data analysis 2 years later, however, showed a significant survival benefit with nivolumab regardless of demographic characteristics which indicates a compensation of the initial impact of age [38]. Consequently, in their analysis of the Checkmate-141 study Saba et al. recommended the use of nivolumab in relapsing or metastatic HNSCC patients regardless of age [39]. The increased PD1 expression on tumor-infiltrating lymphocytes as compared to peripheral lymphocytes is certainly affecting treatment response as well, although there was no correlation with age for this observation.

### Expression of chemokine receptor CCR7

The chemokine receptor CCR7 is responsible for the homing of T cells as well as dendritic cells to lymph nodes and contributes to tolerance induction [40]. Furthermore, CCR7 can activate both B and T lymphocytes and stimulate dendritic cell maturation, and it is known as a classical central memory marker [41].

The increased expression of CCR7 on tumor cells has been shown to be associated with a poor prognosis and with the development of lymph node metastases in HNSCC [11, 42]. While the expression of CCR7 on HNSCC cells is increased, both in cell lines and patients [10], the frequency of peripheral CD8^+^CCR7^+^ T_c_ cells has been found to be decreased in HNSCC patients [12, 13], which in turn is associated with disease recurrence. Generally, CCR7 on peripheral CD8^+^ T_c_ cells is linked to a good outcome for HNSCC patients [12]. We were able to confirm the previous findings of decreased CCR7 expression on CD8^+^ T_c_ cells in tumor patients. Moreover, we detected that the generally higher frequency of protecting CCR7^+^ cells in healthy subjects decreased with age, yet still being higher than in tumor patients (Fig. 5). The observed age-related decrease of CCR7^+^ T_c_ cells may be another result of immunosenescence contributing to the weaker and less responsive immune system in the elderly.

We are well aware that our flow cytometry data should ideally be complemented by additional flow data for FOXP3 and immunohistochemistry. However, in our hands all tumor tissue was urgently needed for the acquisition of fresh TIL. Based on our previously published date, we, therefore, postulate that CD4^+^ CD39^+^ CD25^+^ are all FOXP3^+^.

It has been described repeatedly that frequencies of T_reg_ are elevated in the tumor environment reflecting an immunosuppressive micromilieu [19, 43]. As a result of the fragile immune system in elderly, tumor-induced immune suppression may be less pronounced in aged patients. This hypothesis is supported by the observation that the generally increased frequency of T_reg_ in the tumor microenvironment is significantly decreased in elderly as compared to younger cancer patients (Fig. 1e and f). T_reg_ are supposed to suppress the immune response to tumor-associated antigens. Ambivalently, their presence appears to have diverse prognostic effects depending on the type of cancer. In pancreatic ductal adenocarcinoma, for example, an increased prevalence of T_reg_ has been associated with a poorer prognosis and reduced patient survival [44]. As for HNSCC, however, their presence has been associated with improved survival, particularly in tumors of oropharyngeal origin [16, 45]. The decrease of T_reg_ in tumor tissue of elderly patients could, therefore, indicate a less responsive immune system in this age group which could contribute to a worse outcome of older HNSCC patients. The phenomenon of lower T_reg_ frequencies in tumor tissue of older patients has been stated in a recent melanoma study comparing T_reg_ in the tumor of young and aged mice. Intriguingly, this finding has been confirmed by observations in humans, in which lower T_reg_ frequencies in the tumor tissue were associated with a better response to the PD1 inhibitor pembrolizumab [21]. As elderly patients often do not benefit from chemoradiotherapy [46], treatment with checkpoint inhibitors could possibly present a better alternative or adjuvant therapy option for this patient subgroup.

## Conclusion

Our data demonstrate numerous age-related changes in the immune system in healthy subjects as well as in HNSCC patients. We were able to show a shift towards disturbed immune competence with increasing age mirrored by lower CD8^+^ T cell counts, decreased CCR7 and higher PD1 expression. The weaker immune system of elderly tumor patients could dispense with the need for tumor induced immunosuppression which may in turn be reflected by the lower frequency of tumor infiltrating T_reg_. The number of elderly cancer patients will continuously grow in the future. A detailed knowledge of age-related alterations of the immune system is, therefore, necessary in order to offer an adequate treatment option for this age group.

## Supplementary information

**Additional file 1: Figure S1.** (A) The CD4/CD8 ratio of the subjects were plotted against the age (Spearman Correlation). Each point represents the data of one patient linked the frequency of CD4^+^ T cells and T_reg_, which was shown plotted in (B) for the blood samples of healthy subjects (Pearson Correlation). The total cell numbers of CD8^+^ T cells of the healthy and tumor subjects were plotted against the age (Spearman Correlation) (C). The total lymphocytic numbers of the patients were divided in the five aging groups and plotted. Out of these total lymphocytic numbers the total numbers of CD8^+^ T cells (D), CD4^+^ T cells (E) and the CD39^+^CD25^+^ T_reg_ (F) were plotted. *P* < 0.05 (*); *p* < 0.01 (**); *p* < 0.001 (***). (JPG 5043 kb)

**Additional file 2: Figure S2.** PD1 and CCR7 expression on T cell subtypes. The expression of CCR7 on CD4^+^ T cells (A) for the different aging groups and corresponding plotted in comparison of healthy and tumor blood CD4^+^ T cells (B), and T_reg_ for the different aging groups (C) and the comparison of healthy and tumor blood T cells (D). The expression of PD1 and CCR7 on CD4^+^ T cells, CD8^+^ T cells, and T_reg_ of tumor patients’ blood samples and the co-expression on CD4^+^ T cells, CD8^+^ T cells, and T_reg_ on corresponding TIL in representative density plots (E). All data are plotted showing the mean or the linear regression. *P* < 0.05 (*); *p* < 0.01 (**).

**Additional file 3: Figure S3.** This summary shows the connections between the young and the old subjects in this study. On the left side are mentioned the young volunteers on the right side the old. The young subjects have more CD8^+^ T_c_ cells expressing mainly CCR7 and CD73, while the old subjects have less, expressing more PD1. The young tumor patients have an active immune-system with a strong tumor-induced immune suppression with many T_reg_, while old patients have a senile immune system with a weak immune suppression and less T_reg_.

## Data Availability

The datasets generated and analyzed during the current study are not publicly available due to confidentiality reasons but are available from the corresponding author on reasonable request.

## Abbreviations

ADO: Adenosine
CCR7: C-C chemokine receptor type 7
HNSCC: Head and neck squamous cell carcinoma
mAbs: Monoclonal antibodies
PBL: Peripheral blood lymphocytes
PD1: Programmed cell death 1
TB: Tumor patients’ blood
T_c_: Cytotoxic T cells
T_h_: T helper cells
TIL: Tumor infiltrating lymphocytes
T_reg_: Regulatory T cells
TT: Tumor tissue
