# Supplementary figures and images for: Age-related changes in T lymphocytes of patients with head and neck squamous cell carcinoma
Source: Immun Ageing. 2020 Feb 12;17:3. doi: 10.1186/s12979-020-0174-7 (PMC7017629; doi:10.1186/s12979-020-0174-7)

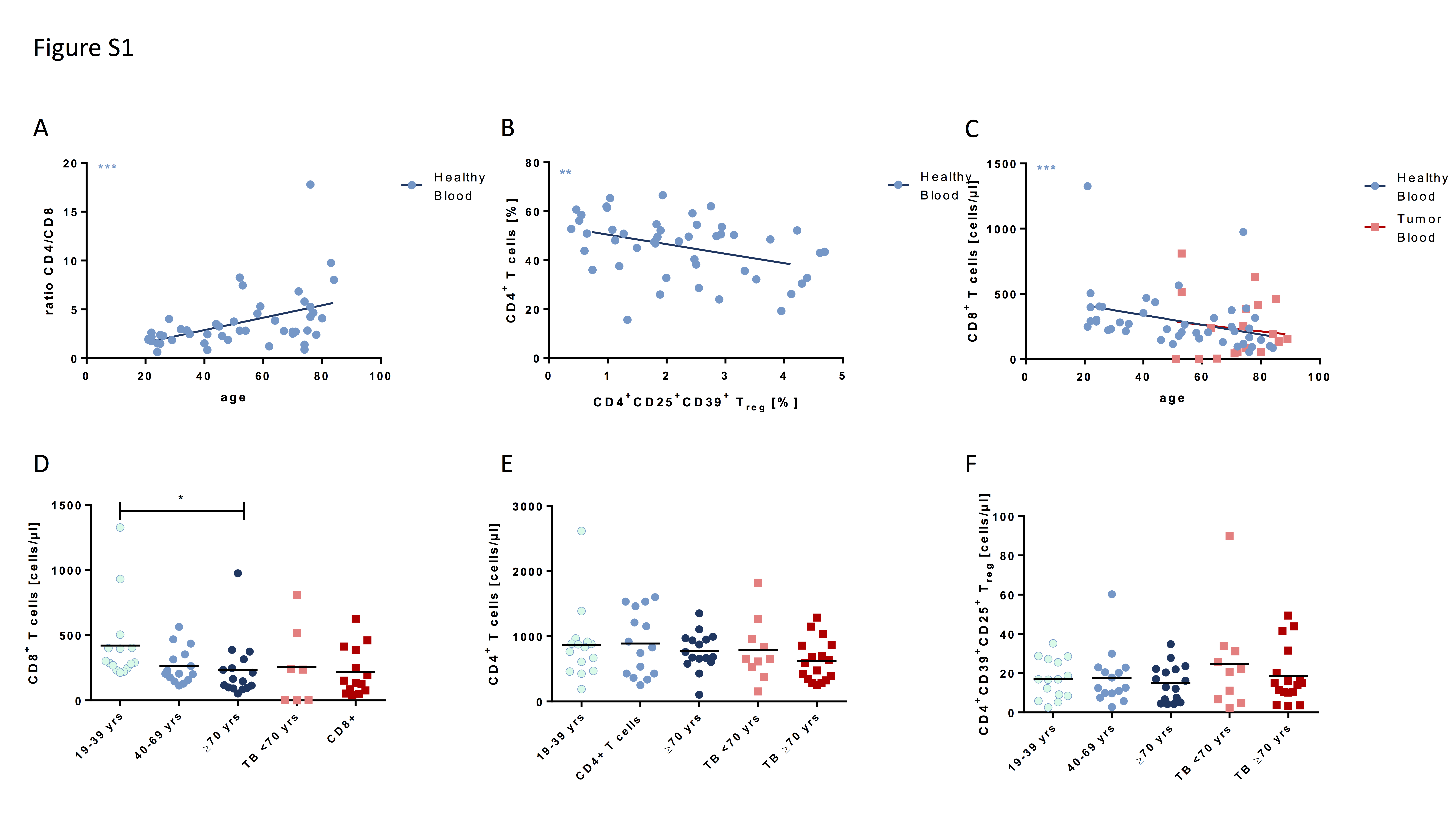

Supplement: Supplementary file 1 — Additional file 1: Figure S1. (A) The CD4/CD8 ratio of the subjects were plotted against the age (Spearman Correlation). Each point represents the data of one patient linked the frequency of CD4+ T cells and Treg, which was shown plotted in (B) for the blood samples of healthy subjects (Pearson Correlation). The total cell numbers of CD8+ T cells of the healthy and tumor subjects were plotted against the age (Spearman Correlation) (C). The total lymphocytic numbers of the patients were divided in the five aging groups and plotted. Out of these total lymphocytic numbers the total numbers of CD8+ T cells (D), CD4+ T cells (E) and the CD39+CD25+ Treg (F) were plotted. P < 0.05 (*); p < 0.01 (**); p < 0.001 (***). (JPG 5043 kb) [file 12979_2020_174_MOESM1_ESM.jpg]

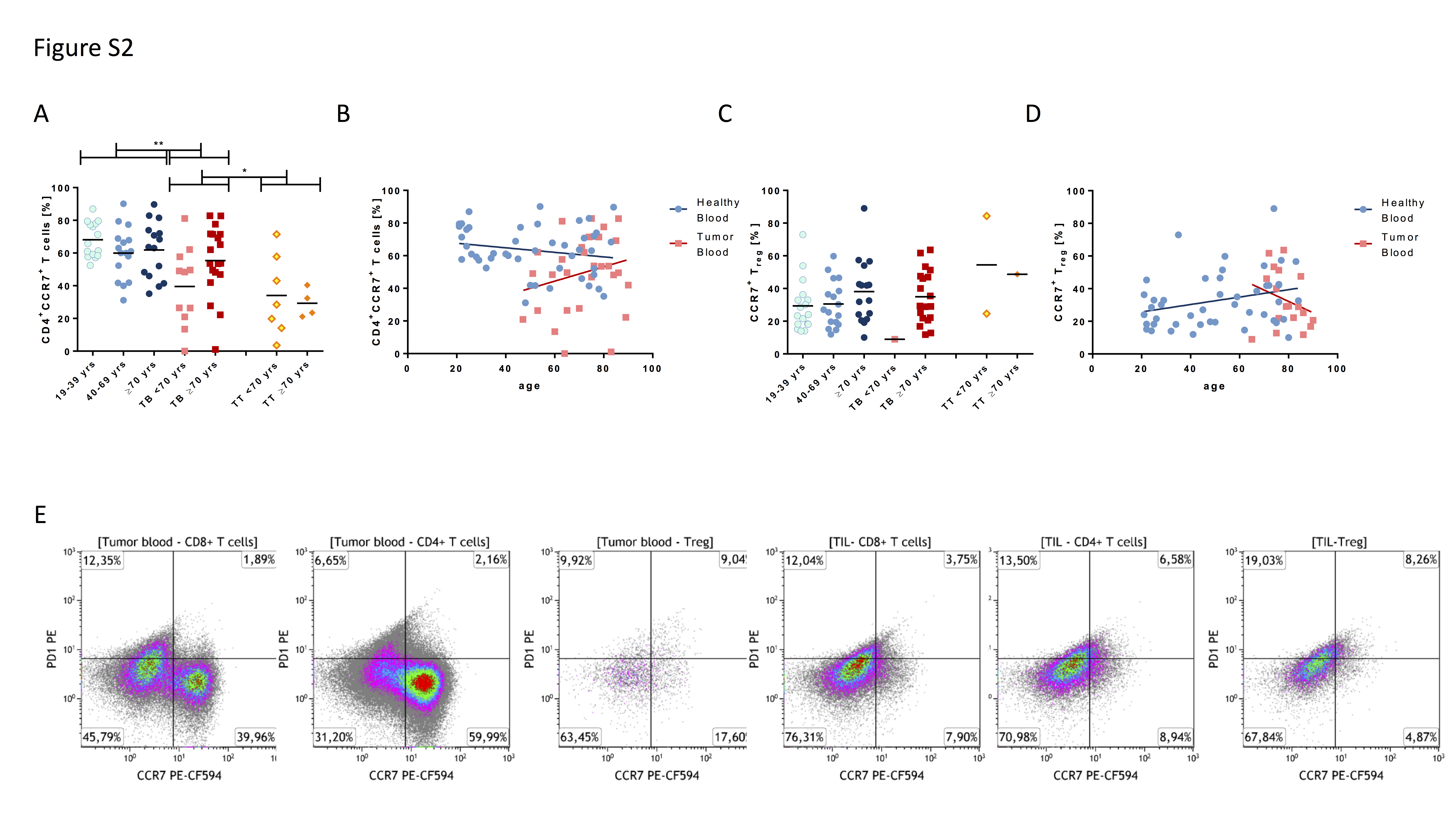

Supplement: Supplementary file 2 — Additional file 2: Figure S2. PD1 and CCR7 expression on T cell subtypes. The expression of CCR7 on CD4+ T cells (A) for the different aging groups and corresponding plotted in comparison of healthy and tumor blood CD4+ T cells (B), and Treg for the different aging groups (C) and the comparison of healthy and tumor blood T cells (D). The expression of PD1 and CCR7 on CD4+ T cells, CD8+ T cells, and Treg of tumor patients’ blood samples and the co-expression on CD4+ T cells, CD8+ T cells, and Treg on corresponding TIL in representative density plots (E). All data are plotted showing the mean or the linear regression. P < 0.05 (*); p < 0.01 (**). [file 12979_2020_174_MOESM2_ESM.jpg]
